# Tumor infiltrating leukocyte density is independent of tumor grade and molecular subtype in aggressive breast cancer of Western Kenya

**DOI:** 10.1186/s41182-017-0059-4

**Published:** 2017-08-04

**Authors:** Rispah T. Sawe, Simeon K. Mining, Ayub V. Ofulla, Kirtika Patel, Bernard Guyah, David Chumba, Jenifer R. Prosperi, Maggie Kerper, Zonggao Shi, Mayra Sandoval-Cooper, Katherine Taylor, Sunil Badve, M. Sharon Stack, Laurie E. Littlepage

**Affiliations:** 1 Department of Immunology, Moi University, College of Health Sciences, School of Medicine, P.O.Box 4606-30100, Eldoret, Kenya; 2 Department of Biomedical Sciences, School of Public Health and Community Development, Maseno University, Kisumu, Kenya; 3 University of Notre Dame, Notre Dame, IN USA; 4 Harper Cancer Research Institute, South Bend, 46617 IN USA; 5 Indiana University School of Medicine, Indianapolis, IN USA; 6 Eck Institute for Global Health, Notre Dame, IN USA; 7 Indiana University School of Medicine - South Bend, South Bend, IN USA

**Keywords:** Tumor infiltrating leukocytes (TILs), Aggressive breast cancer, Breast cancer subtypes, Advanced, African, Kenyan

## Abstract

**Background:**

Tumors commonly are infiltrated by leukocytes, or tumor infiltrating leukocytes (TILs). It remains unclear, however, if the density and type of individual TILs has a direct or simply correlative role in promoting poor prognosis in breast cancer patients. Breast cancer in Kenyan women is aggressive with presentation at a young age, with advanced grade (grade III), large tumor size (>2.0 cm), and poor prognosis. We previously observed that the tumors were predominantly estrogen receptor positive (ER+) but also included both a high percentage of triple negative tumors and also increased immune cell infiltration within the tumors. We used breast tumor tissues from each patient to make tissue microarrays that were then stained for leukocyte and myeloid markers including CD4, CD8, CD20, CD25, CD68, and CD163 using immunohistochemical techniques. The immune cell infiltration into the cancer tissue included increased numbers of macrophages (CD68+), helper T cells (CD4+), and CD25+ lymphocytes compared to benign tissue.

**Results:**

This study characterized the grade, molecular subtypes, and proliferation index of these tumors and determined if TIL density was enriched across any of these factors. We analyzed 49 malignant patient tissue samples for this study. The patient population had a mean age of 51.9 years. The tumors analyzed were heterogeneous by grade: grade I (6%), grade II (47%), and grade III (39%). Most patients presented with large tumors (>2.0 cm) (69%). We classified the tumors into molecular subtypes based on clinical marker expression. Based on this analysis, the molecular subtype distribution was heterogeneous with luminal B (41%), basal/triple negative (TN) (37%), luminal A (14%) and HER2 (8%) breast cancer subtypes. While the basal/TN subtype had a much higher proliferative index (Ki-67+) than did the other molecular subtypes, we did not see a significant correlation between TIL density and either subtype or tumor grade. Therefore, TIL density is independent of molecular subtype and grade.

**Conclusion:**

This study identified a Kenyan patient cohort that develops large, high-grade tumors primarily of the luminal B and basal molecular subtypes. After analyzing the TILs within these tumors, we found that immune cell infiltration of these tumors correlated with increased proliferation but not grade or molecular subtype. Future research is required to determine if the aberrant recruitment of TILs to tumors contributes to cancer progression and response to cancer treatments.

## Background

Ideally, the immune system destroys and inhibits cancer. In contrast, suppression of the immune response can instead promote tumor progression, given the proper context. Recent research has led to increased development and application of immunotherapy, which modulates the immune response, as a cancer treatment [1, 2]. However, the classification of the ability of an immune cell to promote or inhibit cancer progression can be difficult because the immune cell function can be influenced by the microenvironment, which includes other immune cells. Indeed, tumor infiltrating leukocytes (TILs) can be classified as activated, regulatory, or anergic [3].

Both the TIL type and density contribute to the host immune response to breast cancer [3]. In cancer, infiltration of effector T cells correlates with better prognosis [4–6]. In contrast, infiltration of CD25^+^ lymphocytes promotes tumor growth and progression by suppressing the effector function of cytotoxic cells [7]. Also, CD4+ T cells in tumors have cellular plasticity with both anti- and pro-tumor roles [8].

In African patients, breast cancer is diagnosed at a young age and is aggressive, with tumors that are of advanced grade, large in size, and a high rate of triple negative (TN; estrogen receptor negative, ER−; progesterone receptor negative, PR−; and human epidermal growth factor receptor 2 negative, HER2−) [9–11]. The cause of this aggressive cancer has not been elucidated but may be related to the immune response of these patients. Our previous study found that breast cancers of patients in Kenya had increased immune cell infiltration, as indicated by increased recruitment of CD163+ (M2 macrophage), CD25+ lymphocytes, and CD4+ (T helper) cells compared to non-cancer tissue [12].

The immune response in breast cancer patients can have predictive therapeutic value, especially with response to chemotherapy [13]. The presence of or the ratio comparing specific TILs within a tumor may represent immune homeostasis within the tumor and the tumor microenvironment [1]. Although the role and mechanism of the individual TILs in the clinical and biological behavior of tumor are unclear, TILs seem to have predictive value for breast tumors in response to neoadjuvant chemotherapy (NCT) [2, 14]. We previously found increased tumor infiltration of CD25+ lymphocytes in high-grade tumors, which may play a role in suppressing the adaptive immune response leading to poor prognosis in this type of tumors, and modulating these cells could manage aggressive breast cancer types [12]. Other cells, such as myeloid-derived suppressor cells in breast cancer (MDSCs), also may have suppressive action on effector cells by making them immunotherapeutic agents [15].

The variable density and type of immune cells localized to breast tumors may trigger aberrant immune responses in these patients. The heterogeneity of recruited immune cells to the tumors may vary between cancer subtypes, suggesting that different immune cell populations may contribute to tumor suppression or other mechanisms of disease progression or subtype [16]. Alternatively, immune cell recruitment and heterogeneity may be an independent marker of disease progression that may be even more relevant to prognosis and/or treatment. The tumor grade and molecular subtypes of these tumors as well as the level of recruitment of TILs within tumors of certain subtypes were not investigated in our previous study [12]. Therefore, it remains unclear if the TILs that are recruited to the tumors are potential markers that correlate with the molecular subtype or are independent markers of the host immune response. The current study determined if immune cell infiltration into the breast tumors from Kenyan patients might be related to breast cancer subtype and/or tumor grade. Understanding the pattern and function of immune cell infiltration in aggressive tumors of African women is an important strategy to better understand the progression of these tumors. Identifying the significance of the recruitment of these cells in the tumors could form the basis for the identification of markers that represent the host’s immune response.

## Methods

### Study design

This prospective study used consecutively collected breast tumor samples that have been described previously [12]. Briefly, all patients with histologically diagnosed breast cancer who consented to participation in this study, who met the inclusion criteria, and were diagnosed and treated at the MTRH oncology clinic between May 2011 and July 2013 were included in this study (*N* = 68). Patients were included in the study if they had pathologically confirmed breast cancer with no history of chemotherapy. Patients were excluded from this study if they had benign tumors, if they did not consent to participation in the study, if they had a history of cancer prior to the study, or if they used chemotherapy prior to the study. Tissue microarrays generated from these tumors (described below) were stained for the described molecular markers. Of the tumor samples on the tissue microarrays, 49 tumor samples had interpretable staining for all of the indicated markers. Samples from these 49 patients are the samples analyzed and described in this paper. This study adhered to appropriate ethical standards in accordance with approved IRBs from both the University of Notre Dame (IRB Approval # 13-06-1102) and Moi University (IRB Approval # 000655).

### Tissue processing

Specimens were fixed in 10% buffered formalin and then processed through dehydration, xylene, and paraffin infiltration steps in an automatic tissue processor Leica TP 1020 (Leica microsystems Nussloch Gmbh Heidelbeger Sitr. 17-19 D-69226 Nussloch Germany). The blocks were transferred from MTRH to Notre Dame, where the samples were re-embedded into melted paraplast X-tra (McCormick Scientific). Thin sections (3 μm) of tissue samples were cut using rotary microtone (Letz 1512) equipped with disposable blades and stained by hematoxylin and eosin and then analyzed by pathologists.

### Construction of tissue microarrays

We made tissue microarrays (TMAs) of the breast tissue samples [12]. As described, the tissue cores were punched from donor blocks with a 1-mm-diameter stylus and loaded to recipient paraffin blocks. The design of the TMAs made it possible to evaluate the frequency of markers across tumor grades and molecular subtypes from patient tumors. The TMA layout on the recipient TMA blocks was designed in advance to include duplicates of each tissue sample and to organize the tissue samples randomly across the TMAs. Tissue regions were selected and included in the TMA based on the pathology of the tissue determined from the H&E-stained slides of each tissue block. The TMA blocks were constructed with a Veridiam Advanced Tissue Arrayer VTA-110CC. Blocks were sectioned at 3 μm thickness with Leica rotary microtome RM2125. Each of the two TMAs used for the staining had ~100 tissues per block with duplicates of each tissue sample included across the two TMAs.

For breast cancer tissue TMAs, including two or more tissue punches of 1-mm core diameter is preferred over a single punch or larger punch are more consistent and representative of the entire tumor [17, 18].

### Immunohistochemistry

Tissue sections were dewaxed by placing the sections on IHC slides at 60 °C for 30 min followed by processing the samples through three incubations with xylene and then alcohol. After deparaffinization and rehydration in water, the tissues were treated for heat-induced epitope retrieval (HIER). A working solution was prepared by diluting the Envision™ FLEX target retrieval solution (50×) concentration 1:50 in distilled water. Pretreatment module (PT link) was then filled with sufficient quantity (1.5 L) of working solution to cover the tissue sections, and then was set to pre-heat the solution to 65 °C. Tissue sections that were already deparaffinized and rehydrated were immersed to pre-heated Envision Flex target retrieval solution and incubated for 20 min at 97 °C. Then, the sections were left to cool in the PT link to 65 °C.

Each slide rack was removed from the PT link tank and immediately dipped the slides into a jar with a diluted room temperature FLEX TM wash buffer (20×) and left for 1–5 min. Slides were then stained on a Dako autostainer Plus (Dako Colorado, Inc).

The primary antibodies used for our analysis include antibodies that recognize estrogen receptor (ER), progesterone receptor (PR), human epidermal growth factor receptor 2 (HER2), Ki-67, CD4, CD8, CD20, CD68, CD163, and CD25 (Table 1). The secondary antibodies used include EnVision™ Flex+Mouse (Linker), (Dako) SM804; EnVision™ Flex Peroxidase-Blocking Reagent (Dako) SM801; EnVision™ Flex/HRP (Dako) SM802; EnVision™ Flex Antibody Diluent (Dako) DM830; and Hercep Test™ Peroxidase-Blocking Reagent (Dako) SK001. The choice of these markers is supported by the recommendations by the International TILs Working Group 2014, who advocates the scoring of most mononuclear cells but excluding polymorphonuclear leukocytes [16].

**Table 1:** List of primary antibodies

Antibody	Vendor/clone	Pretreatment	Dilution	Control tissue	Detection/linker	Incubation time (minutes)
ER	Dako IR084	TRS high pH	RTU	Breast Ca	Flex	20/20/10
PR	DakoIR068	TRS low pH	RTU	Breast Ca	Flex + M	20/20/20/10
HER2	Dako SK001	Herceptest antigen retrieval		Herceptest	Herceptest	30/30/10
Ki-67	DakoIR626	TRS high pH	RTU	Breast	Flex	20/20/10
CD4	DakoIR649	TRS high pH	RTU	Tonsil	Flex + M	15/10/10/10
CD8	DakoIR623	TRS low pH	RTU	Tonsil	Flex + M	15/15/15/10
CD68KPI	DakoIR609	TRS high pH	RTU	Tonsil	Flex	20/10/10
CD20	DakoIR604	TRS high pH	RTU	Tonsil	Flex	10/10/10
CD25	NC NCLCD25-305	TRS high pH	1:100	Tonsil	Flex + M	15/10/10/10
CD163	Vector Laboratories Inc.VP6017007	TRS high pH	1:100	Bone Marrow	Flex + M	15/10/10/10

### Quality control

A known positive control specimen was used in each staining run to ascertain a proper performance of all the applied reagents. If a positive control specimen failed to demonstrate positive staining, labeling of test specimens were considered invalid hence repeated.

For a negative control, secondary antibody without primary antibody was used to identify any nonspecific staining. If the nonspecific staining was not clearly differentiated from the specific staining of the test specimen that test was considered invalid.

### Image analysis

IHC images were quantified with Aperio Image Analysis Tools software using customized macros from the Color Deconvolution and Cell Quantification algorithms in the Aperio Image Analysis Tools software to analyze each DAB chromogen-stained tissue section. One representative area from each tissue core of the stained tissue sections was manually selected with ImageScope annotation tools and assigned with a position code on the TMA layout for easy identification. After marking all the regions of interest on a TMA IHC slide, analysis with a proper macro was run and the output results were exported as an Excel file for further data organization and processing.

### Statistical analysis

Statistical analysis was completed using GraphPad Prism 6.0. Descriptive statistics were used in summarizing statistical results. Ki-67 and TIL markers were compared across grades and molecular subtype by Kruskal-Wallis followed by Dunn’s multiple comparison tests. Controls and those that did not have either grade data or the appropriate staining information to classify the samples by molecular subtype were excluded in the analysis. Level of significance was set at *p* < 0.05.

## Results

### Grade and molecular subtype determination of Kenyan breast cancer

The purpose of this study was to begin to determine the significance of the immune cell infiltration that we previously described in a prospective study of a breast cancer patient population from Eldoret, Kenya [12]. In this study, we compare the immune cell infiltration with known molecular subtypes and tumor grade to determine if these are interrelated or independent markers. To do this, we used immunohistochemistry quantification data of clinical markers (receptor expression and proliferation marker Ki-67 status), TIL density, and pathology that we described previously [12] (Table 1). We also further investigated the grade and molecular subtype of these tumor samples.

Of our malignant patient tissue sample collection (*N* = 68), 49 of these samples were determined to have been stained and analyzed for expression of all of the markers described in the current study [12]. These 49 samples were used in the analysis for the current study. At presentation of disease, the patients included in our study had an average age of 51.9 years old and median age of 48.5 years old (standard deviation, 17.3) (Table 2).

**Table 2:** Demographics and clinical characteristics of the study group (*N* = 53)

Variable	Number	Percentage
Mean age at diagnosis, 51.9 years		
Median age at diagnosis, 48.5 years		
Standard deviation, 17.3		
Tumor grade		
I	3	6
II	23	47
III	19	39
Not graded	4	8
Tumor size (cm)		
≤2	8	31
2.1–5	11	42
>5	7	27
ER		
Positive	29	59
Negative	20	41
PR		
Positive	19	40
Negative	29	60
HER2		
Positive (3)	7	14
Negative (0–2)	42	86
Ki-67		
<14% (low)	6	12
≥14% (high)	43	88

Most of the patients presented with large tumors (>2.0 cm) (69%) that were primarily ER positive (ER+ 59%), PR negative (PR− 60%), and HER2 negative (HER2− 86%) by immunohistochemistry (Table 2). The tumors were highly proliferative (88%), as determined by a high percentage of Ki-67 positively stained cells. In addition, the grade of the tumors analyzed was heterogeneous: grade I (6%), grade II (47%), and grade III (39%).

We classified each patient tumor into a molecular subtype based on the expression of clinical markers (Table 3). The molecular breast cancer subtypes were identified as described previously [19]. The luminal B (41%) and basal/triple negative (TN) breast cancer subtypes (37%) included the majority of the tumors. Luminal A (14%) and HER2 (8%) subtype tumors were less frequent. This distribution is similar to that seen in other African populations [9, 10]. These data also are comparable to reports from the USA and western countries [20, 21].

**Table 3:** Molecular subtypes

Variable	Characteristics	*N* (%)
Luminal A	ER+ and/or PR+, HER2−, low Ki-67	7 (14)
Luminal B	ER+ and/or PR+, HER2+ (or HER2− with high Ki-67)	20 (41)
Her2	ER− and/or PR-, Her2 + Any Ki-67	4 (8)
Basal/triple negative	ER−, PR−, HER2− and any Ki-67	18 (37)

*ER+/-* estrogen receptor positive or negative, *PR+/-* progesterone receptor positive or negative, *HER2 +/-* human epidermal growth factor positive or negative

### Proliferation varies by molecular subtypes

**Fig. 1:**
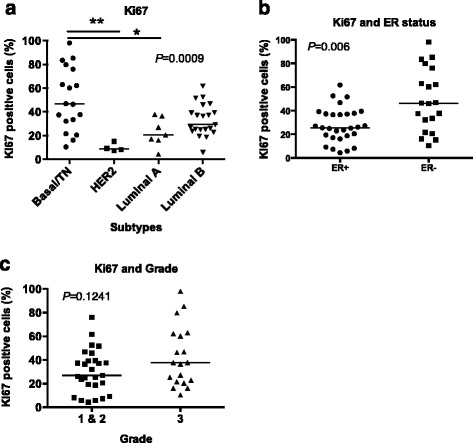
Ki-67 status in Kenyan breast tumors by molecular subtype and grade. Breast cancer tissue samples were stained for Ki-67 and scored for percent Ki-67-positive cells. Ki-67 status then was compared across molecular subtype (**a**) by Kruskal-Wallis (*P =* 0.0009) followed by Dunn’s multiple comparison tests, ER status (**b**) by Mann-Whitney (*P* = 0.006), and grade (**c**) by Mann-Whitney (*P* = 0.1241) (the *P* value is indicated by * and ** for *P* < 0.05 and *P*<0.01, respectively)

In breast cancer, proliferation as marked by high expression of Ki-67 varies by subtype [19, 22–24]. Breast cancers with high proliferation (proliferative marker Ki-67 ≥14%) are associated with poor patient prognosis [25]. We examined proliferation within the tumor subtypes and grades. Ki-67 varied across the molecular subtypes (*p* = 0.0009; Kruskal-Wallis) and was enriched most significantly in the basal/TN molecular subtype and in ER-negative tumors (Fig. 1). This suggests that basal/TN tumors and ER negative had a higher proliferative capacity than did the other subtypes. In contrast, Ki-67 did not significantly vary across grades (*p* = 0.1241) (Fig. 1).

**Fig. 2:**
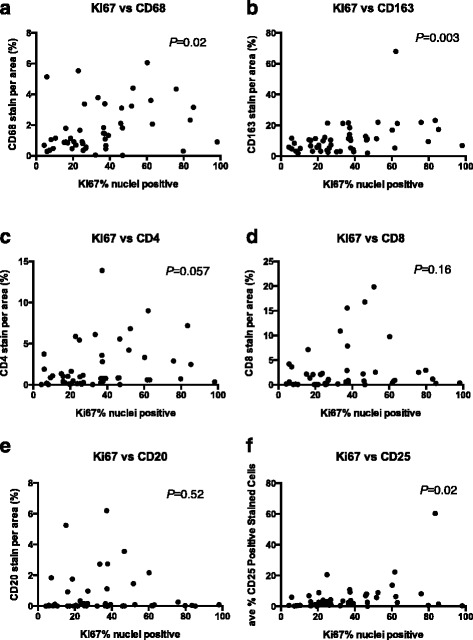
Ki-67 and TIL density correlate in breast tumors from Kenya. Breast cancer tissue samples were stained for TILs, including CD68, CD163, CD4, CD8, CD20, and CD25. Each sample was scored for percentage of positively stained area for the indicated TIL. The Ki-67 positively stained areas/nuclei then were compared across TIL expression levels of CD68 **a**, CD163 **b**, CD4 **c**, CD8 **d**, CD20 **e**, and CD25 **f** by Spearman correlation (*P* = 0.02, 0.003, 0.057, 0.16, 0.52, and 0.0096 with *r* = 0.3419, 0.4023, 0.2741, 0.2140, 0.09378, and 0.3704, respectively)

We next determined if the TIL infiltration densities were related to the proliferative capacity of the tumors by comparing TIL density with Ki-67 proliferation index. We found statistical significance in the correlation between Ki-67 status and CD68, CD163, and CD25 with a similar trend for CD4 (Fig. 2). These data suggest that these immune cells in particular are related to the high proliferative capacity of the tumors.

### Proliferation and tumor infiltrating leukocytes (TILs) in relation to molecular subtypes

**Fig. 3:**
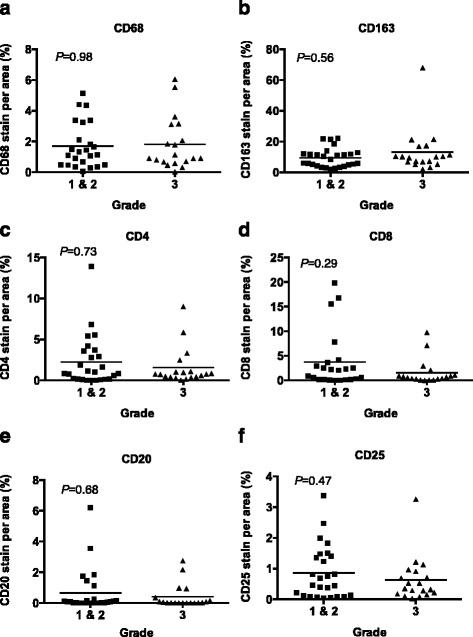
The density of TILs does not differ between tumor grades in breast tumors from Kenya. Breast cancer tissue samples were stained for TILs, including CD68, CD163, CD4, CD8, CD20, and CD25. Each sample was scored for percentage of positively stained area for the indicated TIL. The positively stained areas then were compared across tumor grades 1 & 2 and 3 by Mann-Whitney CD68 **a**, CD163 **b**, CD4 **c**, CD8 **d**, CD20 **e**, and CD25 **f** (*P* = 0.98, 0.56, 0.73, 0.29, 0.68, and 0.47, respectively). The stained area did not significantly vary by grade

**Fig. 4:**
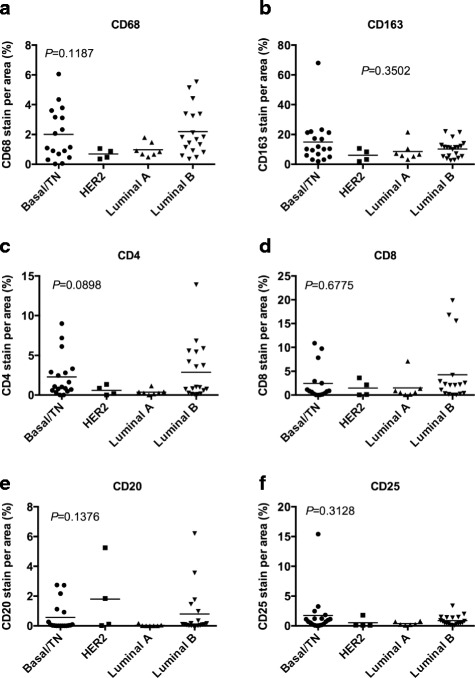
The density of TILs does not differ between molecular subtypes in breast tumors from Kenya. Breast cancer tissue samples were stained for TILs, including CD68, CD163, CD4, CD8, CD20, and CD25. Each sample was scored for percentage of positively stained area for the indicated TIL. The positively stained area then was compared across molecular subtypes basal/TN, HER2, luminal A, and luminal B. The positively stained areas then were compared across molecular subtypes basal/TN, HER2, luminal A, and luminal B by Kruskal-Wallis followed by Dunn’s multiple comparison tests CD68 **a**, CD163 **b**, CD4 **c**, CD8 **d**, CD20 **e**, and CD25 **f** (*P* = 0.1187, 0.3502, 0.0898, 0.6775, 0.1376, and 0.3128, respectively). The stained areas did not significantly vary by molecular subtype

To determine if the immune response actually contributes to the aggressive phenotypes commonly seen in this patient population and that are represented by subtypes, we determined if the levels of immune cell infiltrates correlated with grade and molecular subtypes of breast cancer. We determined if proliferation and individual TILs localized to the tumor tissue were enriched within particular breast cancer molecular subtypes. We found no statistical significance between the density of TILs across grades (Fig. 3) or across the different molecular subtypes (Fig. 4). This suggests that the TIL density does not correlate with molecular subtype or grade and that immune cell infiltration is not simply a surrogate for grade or subtype. Instead, TIL infiltration is an independent marker and another factor, which currently is unknown, must cause the changes in the immune response and correlate with the data.

## Discussion

This prospective study determined that TIL infiltration is an independent marker of breast cancer in these tumors. Our results indicate that TIL infiltration is independent of grade and subtype. In our previous study, we began to characterize the breast cancer seen in patients in Western Kenya. We discovered that these tumors were aggressive with high proliferation and increased immune cell infiltration [12]. In this study, we further classified the grade and molecular subtype of tissue from Kenyan breast cancer patients treated in a regional hospital setting and evaluated TILs as an independent marker of breast cancer in these patients. Using immunohistochemistry molecular markers, we classified these tumors by molecular subtypes. These tumors are heterogeneous with most tumors classified as luminal B (41%) and basal/TN (37%) subtypes. To further study the immune cell infiltration, we determined if the density of individual TILs varied across molecular subtypes and grade or was independent of these. Our results indicate that TIL densities did not significantly correlate with the cancer subtype and tumor grade. Therefore, while immune cell infiltration significantly correlates with malignancy in these tumors, this infiltration is independent of the molecular subtype or tumor grade.

Even though in our study we saw no significant difference in immune cell infiltration in tumors across most molecular subtypes, the individual TILs could hold clinical value as a marker that is independent of subtype and instead applicable across all subtypes but specific to treatment (e.g., chemotherapy-resistant tumors). In breast cancer, infiltration of breast tumors by effector T cells correlates with better prognosis [5, 6, 26]. In contrast, infiltration of CD25^+^ regulatory T cells promotes tumor growth and progression by suppressing the effector function of cytotoxic cells [7]. Also, CD4^+^ T cells in tumors have plasticity and have both anti- and pro-tumor roles [8]. In contrast to our current study, which finds no significant differences in immune cell density between tumor grades, other studies demonstrate differential lymphocyte infiltration in breast cancer tissues based on histological grade, with infiltration by CD4^+^ and CD8^+^ Th1 effector cells in lower grade tumors [3, 27]. However, the immune cell heterogeneity and, more specifically, the ratio of immune cells to other immune cells within a tumor may be critical in identifying any contribution of these cells to patient prognosis.

One limitation of this study could be the use of TMAs rather than the entire tissue sections for TIL and molecular marker analysis. However, we have tried to mitigate this concern by using multiple tissue punches per tissue sample per TMA. When using TMAs of breast cancer tissue, including two or more tissue punches of 1-mm core diameter is preferred and is shown to be more consistent and representative of the entire tumor compared to either a single punch or a larger 2-mm core punch [17, 18]. In our analysis, we included duplicate tissue punches (1-mm core diameter) from each tumor sample to increase the likelihood that the data was representative of the entire tissue. In addition, our analysis scored the number of TILs per cancer tissue and immediately adjacent stroma on a TMA but did not include TMA samples that were predominantly stromal tissue, rather than a cancer-cell-containing tissue. This approach might self-select for a subset of the TIL population and could more appropriately be analyzed by flow cytometry analysis from fresh tissues of single-cell suspensions that are stained and analyzed for TIL markers. However, this option was not available for this study.

The small sample size was a limitation of this study. Our patient population included very few tumors classified as HER2 positive (14%) by immunohistochemistry or as HER2 (8%) by molecular subtype (Tables 2 and 3). For example, due to the small numbers of HER2+ samples, we are unable to draw conclusions about the Ki-67 status of HER2+ tumors within our patient cohort. However, for most other analysis of our data, our patient cohort is sufficiently large to have statistical power. In addition, our breast cancer patient sample size is comparable to other breast cancer studies from Kenya, Uganda, and Tanzania [28–30]. In fact, a large meta-analysis of African breast cancer studies did not find a small study bias in the studies analyzed from sub-Saharan Africa, which includes Kenya [31]. In comparison to these other studies, our study was strengthened by being a prospective study and by not being a convenience sample.

Our data do not rule out the possibility of TILs having a prognostic role in breast cancer progression or a predictive role in response to therapy, since our study did not include patient outcome or therapeutic response data. This study will form the basis for future prognostic studies in African breast cancer patient populations. Since a significant percentage of Kenyan breast cancer patients present with aggressive breast tumors and have poor prognosis, these patients may not benefit from standard therapies and may require alternative therapies, such as immune therapy. For example, the significant population of patients with TN breast cancer has limited treatment options. Triple negative breast cancer (TNBC) is a heterogeneous disease often characterized by aggressive biology and poor prognosis. Efforts to precisely treat TNBC have been compounded by the lack of specific therapeutic molecular targets. Also, in westernized treatment facilities, patients with HER2+ tumors generally are treated with trastuzumab. However, the Kenyan community has limited resources to provide expensive trastuzumab treatment in such a setting where most patients have few treatment options; treatment options will depend on markers that accurately will predict response to treatment. Interestingly, CD8^+^ and CD25^+^ could be such markers, since TILs are associated with improved distant metastases-free survival as well as increased rates of pathological complete response (pCR) after neoadjuvant trastuzumab and chemotherapy in patients with HER2-positive breast tumors [6, 32]. In addition, including Ki-67 in the standard pathological assessment could help to identify and treat aggressive tumors as early as possible.

Since we identify TIL density as being independent of grade and subtype but correlative with proliferation, this study remains consistent with our hypothesis that the TIL infiltrate may be a reasonable marker of immune response, thus suggesting immune modulators as potential therapies that may have efficacy in treating Kenyan breast cancer patients. Future studies will determine the role of immune cell infiltrates in a patient’s response to treatment. Since chemotherapy is the primary line of therapy for treatment-resistant breast cancers, alternative immunomodulatory treatments may be the second-line therapies for these patients. By understanding the immune response generated by the tumors, we may better understand which immunotherapy treatment options would be relevant to treating these tumors. Understanding the pattern and function of immune cell infiltrates in aggressive tumors of African women is a step towards identifying the critical immune cells that could be therapeutic targets in this population and could be suggestive of potential therapeutic strategies aimed at reprogramming the immune response in this patient population.

## Conclusion

In conclusion, we report that in the tumors of our described Kenyan breast cancer patient cohort, immune cell infiltration measured by the cell type and density of tumor infiltrating leukocytes in breast tumors correlates with increased proliferation. However, TIL infiltration does not significantly vary by breast cancer molecular subtypes or by grade. Future studies that collect both additional breast tumor samples as well as longitudinal survival data from patients will be required to determine the prognostic value of our findings.

## Abbreviations

ER: Estrogen receptor
Her2/neu receptor HER2: Human epidermal growth factor 2 receptor
IHC: Immunohistochemistry
MDSCs: Myeloid-derived suppressor cells
MTRH: Moi Teaching and Referral Hospital
NCT: Neoadjuvant chemotherapy
pCR: Pathological complete response
PR: Progesterone receptor
TILs: Tumor infiltrating leukocytes
TMAs: Tissue microarrays
TNBC: Triple negative breast cancer
Tregs: Regulatory T cells
